# Antistreptococcic Serum in Gonorrhœal and Other Infections

**Published:** 1907-02-02

**Authors:** 


### Hospital Clinics.

### ANTISTREPTOCOCCIC SERUM IN GONORRHŒAL AND OTHER INFECTIONS.

### A Discussion at the Therapeutic Society, Apothecaries' Hall, London.

At a recent meeting of the Therapeutic Society a paper on "Some Further Experiences in the use of an Antistreptococcus Serum in Gonorrhœal and other Infections" was read by Dr. J. Porter Parkinson. Dr. Parkinson said that in the few months that had elapsed since Dr. Soltan Fenwick and he read their previous paper before the Royal Medico-Chirurgical Society he was not able to bring forward a large number of new cases, but he could at least show some further encouraging results—results, too, which suggest that the rectal administration of this serum may have even a more extended application than was originally claimed for it. Dr. Parkinson first recapitulated shortly a case taken from the original paper by Dr. Fenwick and himself. A young married woman had been suffering for nine days from what was supposed to be acute rheumatism—she had severe pains and swelling in the right wrist and left knee, and had been feverish and delirious. On admission to the London Temperance Hospital she was delirious, with a hot dry skin, temperature 102° F., pulse 102, and respirations 28 to the minute. The right wrist was swollen and the skin of the dorsum of the hand was reddened, and pitted on pressure. Both knee-joints were swollen and contained fluid, and the right ankle and the dorsum of the right foot were puffy and swollen. The impulse of the heart was feeble, but in the normal position, and a soft systolic murmur was audible over the mitral area and conducted as far as the left anterior axillary line. There was a purulent discharge from the vagina and the uterus was fixed to the left side. The urine withdrawn by a catheter contained a trace of albumen. Though the general condition of the patient was in favour of pyæmia rather than of rheumatism, full doses of salicylate of sodium were ordered, and some of the vaginal discharge was collected for examination. Two days later the temperature had fallen to 100° F. and the pulse rate to 98, but the general condition of the patient was worse and the urine and fæces were passed into the bed. The vaginal discharge was reported to contain numerous gonococci. The salicylate mixture was then omitted and local treatment begun for the gonorrhœa, while a mixture containing quinine and iodide of potassium was given. In about a week the condition appeared unchanged, the temperature varying from 100° to 101° F., and the pulse being about 112. Then the temperature began to rise to 103° F. and the pulse to 120, and the respirations were 40 a minute. The tongue was dry, brown, and cracked, and the complexion had a sallow, earthy hue. The mitral murmur had increased in intensity and a systolic murmur was heard over the aortic area; there were numerous rhonchi and rales over the right lung and pleural friction at the base of the left lung. Six days later the patient appeared moribund and was only kept alive by repeated injections of strychnine. She was quite unconscious, the pulse was feeble, and varied from 130 to 140 a minute. There were signs of œdema of the right lung and of a pleural effusion on the left side. A little turbid fluid drawn from the left side of the chest showed a few gonococci enclosed in the pus cells. In this desperate condition 10 cc. of the polyvalent antistreptococcus serum prepared in the Wellcome Laboratory was injected into the rectum.

The following day the temperature had fallen a degree and a half, but the pulse rate had not diminished. Another rectal injection of the serum was administered. On midday on the third day after the first injection the temperature had fallen to 98° F., the pulse was 108, and the respirations 34 to the minute; the patient was conscious for the first time, the motions were no longer passed into the bed, the aspect of the face had improved wonderfully, and she was able to swallow liquid nourishment with ease. The next day the temperature rose to 100° F., and continued at this point for a fortnight; this was attributed to the pleural effusion, as it gradually declined when the latter became absorbed, and in no way retarded the rapid progress of convalescence. The pain, swelling and stiffness of the affected joints disappeared within a week of the treatment by serum. Altogether three doses of the serum of 10 cc. each were administered within five days. The patient was discharged from the hospital after a stay of eight weeks. At that time the impulse of the heart was in the nipple line, and the mitral murmur though present was less loud than during the illness; the aortic murmur had disappeared. There were signs of adherent pleura at the base of the left lung. The urine still contained a trace of albumen, but the vaginal discharge had quite ceased. This patient was watched in the out-patient department for some months, and continued in perfect health.

This case is an example of three similar ones which have been successfully treated by this method, but as such cases are fortunately not very frequent, Dr. Parkinson has been unable to test the treatment further; in each case the recovery has been as prompt and successful as in the one quoted. In similar cases he suggested for the future an initial dose of 20 cc. of the serum.

The following case, also extracted from the previous paper, shows the result of this treatment in a case of poly-articular gonorrhœal arthritis, usually called gonorrhœal rheumatism. A young married woman was admitted suffering from pain and swelling of the wrists, ankles, and knees. Two years previously she had had an attack of gonorrhœa, followed by severe "rheumatism" for which she was confined to bed for six weeks. She had also had two attacks of "peritonitis." The present illness began a fortnight before admission with a purulent discharge from the vagina and swelling of the wrists and left ankle. The temperature was 101° F. and the pulse rate 88. There was a profuse vaginal discharge containing gonococci in abundance. The wrist joints were swollen and tender, and a puffy swelling extended several inches up the dorsal surface of each forearm. Any movement of the wrist or fingers caused great pain. The left ankle and knee were also affected. The patient was treated by antiseptic vaginal douches, followed by swabbings with a two-per-cent. solution of Protargol, and internally large doses of salicylate of sodium combined with quinine were given. Radiant heat was used to the affected joints. The temperature fell to normal after eleven days' treatment, and the vaginal discharge diminished, but the condition of the joints remained unaffected, and the right knee and ankle became swollen and tender. After another fortnight's treatment by iodide of potassium, etc., there was no improvement in the condition of the joints, though the temperature remained about normal. The treatment as above was now abandoned and 10 cc. of the polyvalent serum was injected into the rectum every other day for three doses. By the sixth day the pain had almost disappeared from the ankles and knees, and the swelling of the wrists and forearms had subsided completely, leaving only a certain amount of stiffness and tenderness on movement. In another week this also had subsided, and the patient was discharged cured a fortnight later.

These cases were selected as types of those treated during the early or experimental stage of the work, and in them the usual methods of treatment were tried before resorting to the use of the serum. They are not, therefore, to be met by the argument that they were of that mild type in which rest in hospital and antiseptic vaginal douches are all that is necessary to effect a cure. Again, it will be seen that the effect of the serum is so prompt that it is impossible not to believe that the improvement is due to its use. Dr. Parkinson went on to show the value of the treatment in various types of joint affections associated with, or caused by, gonorrhœal disease. First among these may be mentioned the *arthralgic* type in which there are wandering pains about the joints without redness or swelling. This variety may persist for years.

The author recently saw a gentleman, aged over sixty, who had had gonorrhœal infection when a young man, followed by what was considered to be "rheumatism" three years later, during which condition, however, the urethral discharge reappeared. The attack crippled him for some weeks; it was followed by stiffness of the joints, and this had increased considerably during the last ten years. It frequently necessitated him mounting stairs on his hands and knees, and caused pain and difficulty in getting up from a low chair. The joints were not obviously enlarged or the ends of the bones thickened. Five or six months ago he was treated by three rectal injections of serum on alternate days, with the result that the pain and stiffness gradually improved, and for the last few months he has been able to walk and use his limbs freely and without pain. He now describes himself as better than he has been for many years. Dr. Parkinson had a letter from this gentleman on November 22, in which he says: "I have been absolutely free from everything connected with the rheumatic annoyance; I was nearly three months getting rid of the effects of previous annoyances, though all rheumatism proper ceased at the end of July. The serum seems to have been an absolute success in my case."

Another case of chronic arthritis was that of a charwoman, aged 52, who has suffered from "chronic rheumatism" for ten years; this has successively affected the feet and ankles, elbows, hands, and knees; the trouble was at first only at intervals, but since November 1905, became constant, and about that date she came under Dr. Parkinson's care as an out-patient. There was some thickening about, and effusion into, the knee-joints. She was treated with iodide of potassium and quinine, as well as locally, but with only moderate improvement. On November 5 she complained of much pain in the right metacarpo-phalangeal joints, and there was much peri-articular swelling extending up the dorsum of the hand. She was given 20 cc. of the serum per rectum, and three days later the pain and swelling had quite disappeared. On November 15, however, the left hand and wrist became similarly affected, and another injection was given with equally rapid improvement. She was seen twice in December, and has had no more pains of any kind. This patient had never had rheumatic fever, but used to suffer severely from leucorrhœa.

Chronic hydrarthrosis, usually monarticular, and especially involving the knee-joint, seems often to be associated with a prolonged gleet or stricture of the urethra. The effect of the serum does not seem to be as satisfactory as in other varieties. The fluid may disappear from the joint, but there is frequently considerable stiffness and extra-articular swelling which may persist for some time just as in cases not treated by the serum method.

The *bursal and synovial form* of the disease, which attacks chiefly the sheaths of the tendons, bursæ, etc., may occur along with more serious forms in which endo- and peri-carditis, pleurisy, and evidences of a general affection are present, and in these the effect of the serum is remarkable; the first case related is an example of this form. In other cases, the disease is more limited to the bursæ and sheaths of the tendons, and though in some rapid improvement has resulted from the treatment, in others the disease has appeared to run its course uninfluenced by it. In these latter cases there has usually been a urethral stricture.

In the other varieties, such as the *polyarthritic*, the *septicæmic*, and the *acute arthritis* of one or two joints, the polyvalent serum appears always to effect a rapid and permanent improvement.

The fact that in various cases treated by Dr. Fenwick and Dr. Porter Parkinson the urethral discharge disappeared along with the other phenomena, led them to try the effect in cases of acute gonorrhœa. Their experience has been limited by paucity of material, but in those treated the urethral discharge has disappeared within a week, when treated solely by serum rectal injections. Mr. Paterson, surgeon at the Lock Hospital, states, however, that in acute gonorrhœa the treatment is not very efficacious, but he corroborated the previous evidence as to its value in certain cases of gonorrhœal arthritic infections.

Here the author drew attention to the interesting fact, mentioned in the previous paper, that Dr. Dowson, the director of the Wellcome Laboratories, has found that while the gonococcus grows readily in a medium to which the antigonococcic serum had been added, it failed to grow in the presence of the polyvalent antistreptococcic serum, and this observation he has confirmed by further experiments.

In some cases of purpura *hæmorrhagica* the rectal use of this serum is followed by immediate improvement, as witness the following extreme case.

A warehouse porter, aged 22, had been the subject of pulmonary phthisis for six months. Two days before admission he felt ill, red spots appeared on the legs and trunk, and blood began to issue from the mouth; the next day blood appeared with the urine and the stools. On admission he was very weak and anæmic, the temperature was 97.2° F., pulse 106, and respirations 34. The muco-purulent sputum contained numerous tubercle bacilli, and there was evidence of consolidation of the upper part of the left lung, and moist rales were heard over both bases.

Over the whole of the body and limbs were purpuric spots varying in size from a pin's head to a sixpence, and here and there larger ecchymoses. The tongue, lips, gums and palate were pale and covered with petechiæ, and blood constantly dripped from the mucous membranes of the mouth and nose. The urine was dark with blood and became almost solid on boiling; on standing it deposited nearly half its bulk of red blood corpuscles. The stools were loose, consisting almost entirely of altered blood. A blood-count showed only 60 per cent. of red blood corpuscles. The patient got rapidly worse and the bleeding from the mucous membranes and internal organs became more profuse. The pulse became feeble and intermittent, and partial consciousness alternated with muttering delirium. Some œdema of the legs appeared. At this stage the patient appeared to be inevitably dying, and though a good result was hardly to be expected, 10 cc. of polyvalent serum were injected into the rectum, and this dose was repeated at 6 P.M. The next day the man was much better, the pulse was 96 and of higher tension, the bleeding from the gums had almost ceased, and the urine and stools contained much less blood. The next day the temperature had risen to 99° F., and the pulse declined to 90. All bleeding had ceased from the gums and bowel, and the urine was only slightly smoky. Two more injections were given, and five days after admission the patient appeared to be convalescent. The purpuric eruption rapidly faded, and three weeks later the patient was discharged well except for his pulmonary trouble. Cultures from the blood exhibited no bacterial growth. Since this case two more patients suffering from severe purpura hæmorrhagica have been treated in the same way and with a rapidly successful result.

While, therefore, not suggesting that this serum is a specific for all such cases, it seems only logical to consider that it is worthy of further trial, especially in view of the fact that no harmful results follow its use.

Dr. Parkinson has recently tried the serum in cases of *fibromyositis*, such as lumbago and the painful swellings in the deeper muscles attached to the scapula, and has had a fair proportion of cases in which the treatment has been most successful, but it is difficult to be quite sure of the effect of any treatment in conditions like these, which are sometimes very fugitive and may disappear spontaneously; and also when, as is the case of hospital patients, they may be lost sight of after one or two attendances.

The following notes were taken from such a case. A married woman, aged 40, had suffered for four-and-a-half years from chronic pains, chiefly in the muscles, intensified on movement and specially marked after a rest. She had also occasional pain and stiffness in the right hip-joint. On and off there has been leucorrhœa. There was nothing abnormal to be made out on examination, and the heart was normal. One injection of 20 cc. of the serum relieved the muscular and arthritic pains, and the leucorrhœa ceased within a week.

The pathology of so-called chronic rheumatism of joints and muscles is still very obscure, though there is but little doubt of their connection with certain microbic diseases of which gonorrhœa is one. When these organisms get into the blood-stream they may lodge in fibrous structures and there produce a local reaction with inflammatory proliferation of tissue, which accounts for the racking pains present in chronic muscular rheumatism.

At present it seems impossible to determine which organism is the causal factor in a particular case, but these therapeutic experiments show that a fair proportion yield rapidly to serum treatment even in the absence of any other medication.

As the polyvalent serum is prepared from 43 different strains of streptococci it may be certainly designated a "shot-gun remedy," but till our knowledge is greater it appears to be a useful weapon, and we do not yet know to which of the different strains of micro-organisms the effect is due.

Dr. Parkinson hoped there would be a more extended trial of the remedy, as the results already obtained seemed amply to justify its further use.

Dr. Hope read a paper on the use of anti-diphtheric serum in tubercular disease. He came to the conclusion that while diphtheria antitoxin was an aid to the treatment of the disease in its early stages, it was of no value in the chronic and advanced stages of phthisis.

Dr. William A. Potts, Birmingham, said it had been recognised for some time that diphtheria antitoxin was an excellent remedy in many cases of bad throat where the poison was not necessarily the diphtheria bacillus, and where the mischief was due to other organisms. It is not necessary to wait until the bacterial investigation of the swab has been made; the antitoxin is extremely useful in such cases, and there is no inherent improbability in its being useful in such cases as Dr. Hope had described. He desired to know whether in the investigations any trouble had arisen from the intense irritation caused by the injection of the serum. He had a lively recollection in his own person of such a phenomenon which lasted three days, and caused him to hesitate to use it again. No remedy seemed to be able to relieve the irritation.

Dr. Herbert French asked whether the anti-streptococcic injection had been used with the ordinary enema tube, and whether it had been diluted or undiluted. He referred to the use of the opsonic index as a means of testing the value of the treatment. If it did good, one would imagine an increase of the opsonic index to occur. In healthy people the injection of anti-diphtheric serum caused no change in the tuberculo-opsonic index. In tuberculous people inoculated with anti-diphtheric serum some change might occur. If it did good it would be proved by a rise in the opsonic index, and in that way one would have a guide as to when the injection should be repeated, say, in ten days or two weeks. He had met a medical man from Australia some time ago with notions of the value of anti-diphtheric serum in tuberculous disease who could not get his book published in London. There was apparently great value obtained in giving these serums per rectum. Sir Dyce Duckworth had said that in infectious endocarditis much benefit was obtained by giving the serum per rectum. In a bad case of erysipelas in Guy's Hospital the patient was relieved within 24 hours of giving one phial of 10 c.c. per diem per rectum. The treatment was continued for three days. The dangerous condition recurred when the injection of serum was suspended. It was resumed in 10 c.c doses with good results. In that patient there was no irritation.

Dr. Bentley, Limpsfield, Surrey, had tried the remedy in several cases of consumption. Three of the cases were too far advanced. One case had tubercle of the larynx, with thick cushion-like epiglottis, and one lung was affected. He treated it once a fortnight for six weeks with anti-diphtheritic serum. The larynx improved and the lung disease did not extend. The patient felt better, took more exercise, got up hills better, and was beginning to gain weight. He kept up the injections until the patient began to be quite intolerant and exhibited irritability towards the serum; it was therefore stopped for three months. The other lung showed some signs of breaking down, and the feeling of lassitude returned. The injections were renewed, and the patient began to improve at once.

Dr. Ross, Perth, said he had made a study of the subject in the way of reading, but could find no authority whose advice would lead him to give the anti-diphtheric serum by the mouth, although some authorities held that it was equally efficient given in that way. If it should happen that diphtheria antitoxic serum could be given by the rectum or by the mouth, it would be very important for the general practitioner who might have cases requiring repeated injections at long distances apart; if one could confidently say that it would act as well by the mouth it would save many a long journey in country practice. Some definite ground, however, is required to proceed upon, but if it could be devised the mouth administration would be a great advantage. At the last meeting of the British Medical Association the question was rather shirked, and none of the superintendents of the London fever hospitals appeared to care to risk the administration of it by the mouth or rectum.

Dr. Porter Parkinson, in reply, said that in most of the cases of single-joint infection he had not thought it advisable to put a needle into the joint, the infusion had disappeared with strapping, and it was not necessary to remove the fluid for treatment. With regard to the kind of serum used, the only one which he had employed was the polyvalent anti-streptococcic serum prepared by Burroughs Wellcome and Co. at their laboratory, which consisted of 43 different strains of organisms. He had no information concerning the effect of the gonococcus itself, but the gonococcus grew quite well in a culture to which anti-gonococcic serum had been added. The first case treated was absolutely a shot in the dark, and he was surprised to hear the day following that the patient was still alive. The results in chronic rheumatism were good. It may be that in the cases treated the gonococci may have been latent for years. These chronic cases of articular rheumatism may be produced by the poison. If the serum is useful in some kinds of rheumatism it may be useful in others. Irritation of the skin, Dr. Porter Parkinson thought, was only produced when the injection was made under the skin. In the case he had to deal with it had been injected into the rectum by means of an ordinary glass syringe with a catheter, the antitoxin being poured into the syringe in the usual way, and its own weight being sufficient to secure its entrance. No bad effects have ever happened. The method is not new, but has been proceeding for six or seven years. It was first alluded to by himself in a letter to the *British Medical Journal*, six years ago: since that time many persons have used it and published cases on it. In cases of diphtheria, rapidity of application is the principal thing, and so it is essential to make the injection under the skin in that disease. In one case he had injected 4,000 units by the rectum. He supposed that the child must have been ill for 36 hours before he saw it. The child lived, did fairly well, and eventually recovered. But he could not advise this method in diphtheria, as absorption is probably more rapid when given subcutaneously.

